# Genomic and Transcriptomic Analysis of Bovine *Pasteurella multocida* Serogroup A Strain Reveals Insights Into Virulence Attenuation

**DOI:** 10.3389/fvets.2021.765495

**Published:** 2021-11-10

**Authors:** Li Zhan, Jiaqi Zhang, Boyu Zhao, Xintian Li, Xiqing Zhang, Renge Hu, Emad Mohammed Elken, Lingcong Kong, Yunhang Gao

**Affiliations:** ^1^College of Animal Science and Technology, Jilin Agricultural University, Changchun, China; ^2^Marine College, Shandong University, Weihai, China; ^3^Animal Production Department, Faculty of Agriculture, Al-Azhar University, Cairo, Egypt; ^4^The Key Laboratory of Animal Production, Product Quality and Security, Ministry of Education, Jilin Agricultural University, Changchun, China

**Keywords:** *P. multocida* serogroup A, complete genome sequencing, transcriptomic sequencing, gene islands, virulence gene

## Abstract

*Pasteurella multocida* is one of the primary pathogens of bovine respiratory disease (BRD), and causes huge losses in the cattle industry. The Pm3 strain was a natural isolate, which is a strong form of pathogen and is sensitive to fluoroquinolones antibiotics. A high fluoroquinolone resistant strain, Pm64 (MIC = 64 μg/mL), was formed after continuous induction with subinhibitory concentration (1/2 MIC) of enrofloxacin, with the enhanced growth characteristics and large attenuation of pathogenicity in mice. This study reports the whole genome sequence and the transcription profile by RNA-Seq of strain Pm3/Pm64. The results showed an ineffective difference between the two strains at the genome level. However, 32 genes could be recognized in the gene islands (GIs) of Pm64, in which 24 genes were added and 8 genes were lost. Those genes are involved in DNA binding, trehalose metabolism, material transportation, capsule synthesis, prophage, amino acid metabolism, and other functions. In Pm3 strain, 558 up-regulated and 568 down-regulated genes were found compared to Pm64 strain, from which 20 virulence factor-related differentially expressed genes (DEGs) were screened. Mainly differentially transcribed genes were associated with capsular polysaccharide (CPS), lipopolysaccharide (LPS), lipooligosaccharide (LOS). Iron utilization, and biofilm composition. We speculated that the main mechanism of virulence attenuation after the formation of resistance of Pm64 comes from the change of the expression profile of these genes. This report elucidated the toxicity targets of *P. multocida* serogroup A which provide fundamental information toward the understanding of the pathogenic mechanism and to decreasing antimicrobial drugs resistance.

## Introduction

In recent years, the high incidence of bovine respiratory diseases (BRD) has seriously affected the cattle breeding industry worldwide, and these diseases mainly occur in fattening cattle and calves during long distance transportation, and cause a lack of energy and appetite, accompanied by cough, runny nose, and dyspnea (1). In North America, BRD accounts for ~75% of all disease incidence and 50% of all mortality in cattle farms, with an even higher prevalence (nearly 90%) in calves (2).

Bacterial pathogens which are associated with BRD include *Pasteurella multocida, Mannheimia haemolytica, Mycoplasma bovis*, and *Histophilus somni* (3). Among these bacteria, *Pasteurella multocida* (*P. multocida*), a pathogenic gram-negative bacterium, has been divided into three subspecies, five capsular serogroups, and 16 serotypes. *P. multocida* serogroup A is commonly isolated from both enzootic calf pneumonia of young dairy calves and shipping fever of weaned and stressed beef cattle (4). In Belgium between 2016 and 2018, analysis of the cattle samples from 128 BRD outbreaks found *P.multocida* with a detection rate of 89.1% (5). Several researchers confirmed that *P. multocida* serogroup A was the primary pathogen causing BRD in China, and in our previous study, we found it has a higher risk of fluoroquinolone resistance during antibiotic therapy (6, 7).

After acquiring antibiotic resistance, bacteria are prone to incurring fitness costs, which are manifested in survival inhibition, transformation rate retardation, and virulence attenuation in resistant strains compared with wild type strains without antibiotic treatments (8, 9). But the cost is not absolute; some bacteria do not experience this phenomenon, while others have a compensative evolution to maintain their competitive advantage and levels of resistance without antibiotic pressure (10–12). It blows a hole in the theory that reducing the use of antibiotics can reduce antibiotic resistance. Developing targeted drugs at the molecular level to coordinate antibiotic use to control antibiotic resistance and virulence factors may lead to more effective control.

During the evaluation of antimicrobial susceptibility test and antibiotic resistance risk of *P. multocida* isolates in our research, one *P. multocida* serogroup A isolate (Pm3) showed a strong virulence to mice and resistance developed rapidly with the increase of fluoroquinolones *in vitro*. Hence, a fluoroquinolone-resistant strain, Pm64, was obtained from fluoroquinolone-sensitive strain Pm3 induced by enrofloxacin at a subinhibitory concentration (Increasingly 1/2 MIC). And we found that the virulence of the Pm64 strain to enrofloxacin decreased significantly. This study aimed to further analyze the mechanism of toxicity attenuation of *P. multocida* serogroup A with the development of fluoroquinolone drug resistance. In this study, the difference between *P. multocida* Pm3 and Pm64 strains were compared on genomic and transcriptome levels. We hypothesized that genes and gene expression patterns investigations will show the differences between the two strains which may elucidate the underlying molecular virulence attenuation mechanisms of *P. multocida*. We found several candidate genes that may be highly important to the virulence attenuation of *P. multocida* serogroup A strains and may facilitate the design of new and improved vaccines and target drugs to overcome the rapid growth of antibiotic resistance.

## Materials and Methods

### Bacterial Strains and Culture Conditions

*P. multocida* serogroup A Pm3 strain used in the present study was isolated from the nasal cavity of cattle with BRD in Henan China. Previous studies have identified the molecular type of Pm3 (7). The Pm64 strain was induced from Pm3 strain by gradually increasing subinhibitory concentration (1/2 MIC) enrofloxacin in a liquid culture environment. Every 12 h was recorded as one generation, and the growth was observed to determine whether enrofloxacin concentration doubled or not. All the strains were streaked on Brain Heart Infusion (BHI) agar plates (Qingdao Hope Biol-Technology Co., Ltd., Qingdao, China). One colony of each strain was inoculated into 5 mL BHI broth at 37°C with shaking (160 rpm).

### Median Lethal Dose (LD_50_) Determination

The LD_50_ of Pm3 and Pm64 strains were determined by the modified Kirschner method, and the colony count of each strain was carried out (13). Then, four graded doses of 1 × 10^6^-1 × 10^9^ and 1 × 10^11^-1 × 10^14^ colony-forming units (CFU)/mL of the two strains were set through the pre-experiment. Eight mice, with equal numbers of males and females (20 ± 3 g), were randomly selected from each group. Mice by intraperitoneal injection of 0.2 mL bacterial suspension of different concentration gradients and the control group was injected with equal pH 7 phosphate buffer solution (PBS). The mice in each group were kept isolated, the death rate was observed for 3 days, and the LD_50_ was counted and calculated (14). The data were statistically analyzed using SPSS (19.0) software. The lungs of dead mice were fixed in formalin, embedded in paraffin, and then stained with H&E. Observe the pathological changes under a microscope. All the methods were carried out in accordance with the US NIH guidelines and protocols for laboratory animal use and proper care, approved by the Animal Care and Use Committee of Jilin Agricultural University.

### Growth Curves Measurement

Pm3 and Pm64 strains were aerobically subcultured twice in the BHI medium. 4% cultures were selected to inoculate fresh BHI medium for monitoring growth at 37°C with shaking(160 rpm). The growth rates of Pm3 and Pm64 cultures were plotted by recording OD changes at 0, 2, 4, 6, 8, 10, and 12 h.

### Whole Genome Sequencing

Genome DNA of Pm3 and Pm64 was extracted using a genomic DNA kit (A&A Biotechnology, Gdansk, Poland). The harvested DNA was detected by the agarose gel electrophoresis and quantified by Qubit^®^ 2.0 Fluorometer (Thermo Scientific). Then the large fragments of DNA were recovered by Blue Pippin automatic nucleic acid fragment recovery system, and repaired. Barcode was added by PCR-free method of EXP-NBD104 kit (Oxford Nanopore Technologies Company). The size of fragments was detected by AATI automatic capillary electrophoresis instrument, and the samples were isomolar mixed.After that the SQK-LSK109 connection kit was used to link the adapter. The Nanopore PromethION platform Libraries for sequencing were constructed with an insert size of 10 kb. Next, Sequencing libraries were generated using NEBNext^®^ Ultra™ DNA Library Prep Kit for Illumina (NEB, USA). The whole genome of Pm-3 and Pm-64 was sequenced using Nanopore PromethION platform and Illumina NovaSeq PE150 at the Beijing Novogene Bioinformatics Technology Co., Ltd. Genome sequences were assembled using Unicycler to combine PE150 data and Nanopore data, then the reads were compared to the assembled sequence, the distribution of sequencing depth was couned, whether the assembled sequence is a chromosomal sequence or a plasmid sequence was distinguished according to sequence length and alignment, and it was checkedwhether it was a circular genome. The Pm3 DNA sequence was deposited in GenBank with the accession number CP081486 (BioSample SAMN20842242). The Pm64 DNA sequence was deposited in GenBank with the accession number CP081487 (BioSample SAMN20845833).

### Genomic Analysis

Related coding genes were retrieved using the GeneMarkS program and the interspersed repetitive sequences were predicted using the RepeatMasker (perl 5.8.0). The tandem Repeats were analyzed by the TRF (Tandem repeats finder). Genomics Islands were predicted using IslandPath-DIOMB program. Gene function of the whole genome sequences was subsequently annotated with GO (Gene Ontology), KEGG (Kyoto Encyclopedia of Genes and Genomes), COG (Cluster of Orthologous Groups of proteins), and NR (Non-Redundant Protein Database). The secretory proteins were predicted by the Signal P database, and the prediction of Type I-VII proteins secreted by the pathogenic bacteria was based on the EffectiveT3 software. Pathogenicity and drug resistance were analyzed using the PHI (Pathogen Host Interactions), VFDB (Virulence Factors of Pathogenic Bacteria), and ARDB (Antibiotic Resistance Genes Database).

### RNA Sequencing

Total RNA of Pm3 and Pm64 bacterial samples were extracted using Trizol reagent (Invitrogen Life Technologies, USA), following the manufacturer's protocol, each treatment was conducted in triplicate. RNA degradation and contamination of samples was monitored on 1% agarose gels. RNA integrity of samples were assessed using the RNA Nano 6000 Assay Kit of the Bioanalyzer 2100 system (Agilent Technologies, CA, USA). After the RNA quality testing, the ribosomal RNA (rRNA) in total RNA was removed to obtain mRNA. Subsequently, the obtained mRNA was broken into short fragments randomly by fragmentation buffer, and the library was built in a chain-specific way (15). After that, Qubit2.0 Fluorometer was used for preliminary quantification, and the library was diluted to 1.5 ng/ul. Then the Agilent 2100 bioanalyzer was used to detect the insert size of the library. qRT-PCR was used to accurately quantify the effective concentration of the library (>2 nM) when the insert size meets expectations (StepOnePlus Real-time PCR Systems, Thermo Science). Finally, samples were sequenced on the Illumina Novaseq platform, and 150-bp paired-end reads were generated. The sequencing data were deposited in GenBank, with accession number GSE182406.

### RNA-Seq Analysis

Clean data (clean reads) that all the downstream analyses were based on were obtained from Raw data (raw reads) by removing reads containing adapter, reads containing ploy-N, and low quality reads. At the same time, quality scores Q20, Q30, and GC content of the clean data were calculated. Reads were mapped onto the genes of *P. multocida* 36950. Both building index of the reference genome and aligning clean reads to reference genome used Bowtie2-2.2.3 (16). HTSeq v0.6.1 was used to count the read numbers mapped to each gene. And then FPKM (Fragments Per Kilobase of transcript per Million mapped reads) of each gene was calculated based on the length of the gene and read count mapped to the gene. Differential expression analysis (DEGs) of two strains was performed using the DESeq2 (17). Genes with an adjusted log2(FoldChange)| > 0 & Padj < 0.05 found by DESeq2 were assigned as differentially expressed. Gene Ontology (GO) enrichment analysis of differentially expressed genes was implemented by the GOseq R package, in which gene length bias was corrected. And the statistical enrichment of differential expression genes in KEGG pathways (http://www.genome.jp/kegg/) was tested using KOBAS software.

### Quantitative Real-Time PCR

Take 1ml of logarithmic growth phase bacterial liquid and use EZ gene bacterial RNA kit (BIOMIGA, California, USA) for total RNA extraction. cDNA was synthesized using PrimeScript™ RT reagent Kit with gDNA Eraser (TaKaRa, RR047A, Japan), and qRT-PCR was performed using TB Green^®^ Premix Ex Taq™ II (Tli RNaseH Plus) (TaKaRa, RR820A, Japan). The premixing system is 20ul (10 μL 2× TB Green Premix Ex Taq II, 0.8 μM for each primer, 2 μg cDNA, add water to 20 μL). The PCR reaction conditions were as follows: 95°C for 30 s, 45 cycles at 95°C for 5 s, and 55°C for 60 s). Each target gene was individually normalized by the reference gene 16S rRNA using the quantification method 2^−ΔΔ*Ct*^ (18). The specific primers were designed according to the reference sequences in NCBI with Primer-BLAST and the qRT-PCR primer sequences were listed in Supplementary Table 1.

## Results

### Induction and Biological Characteristics of Pm64

**Figure 1:**
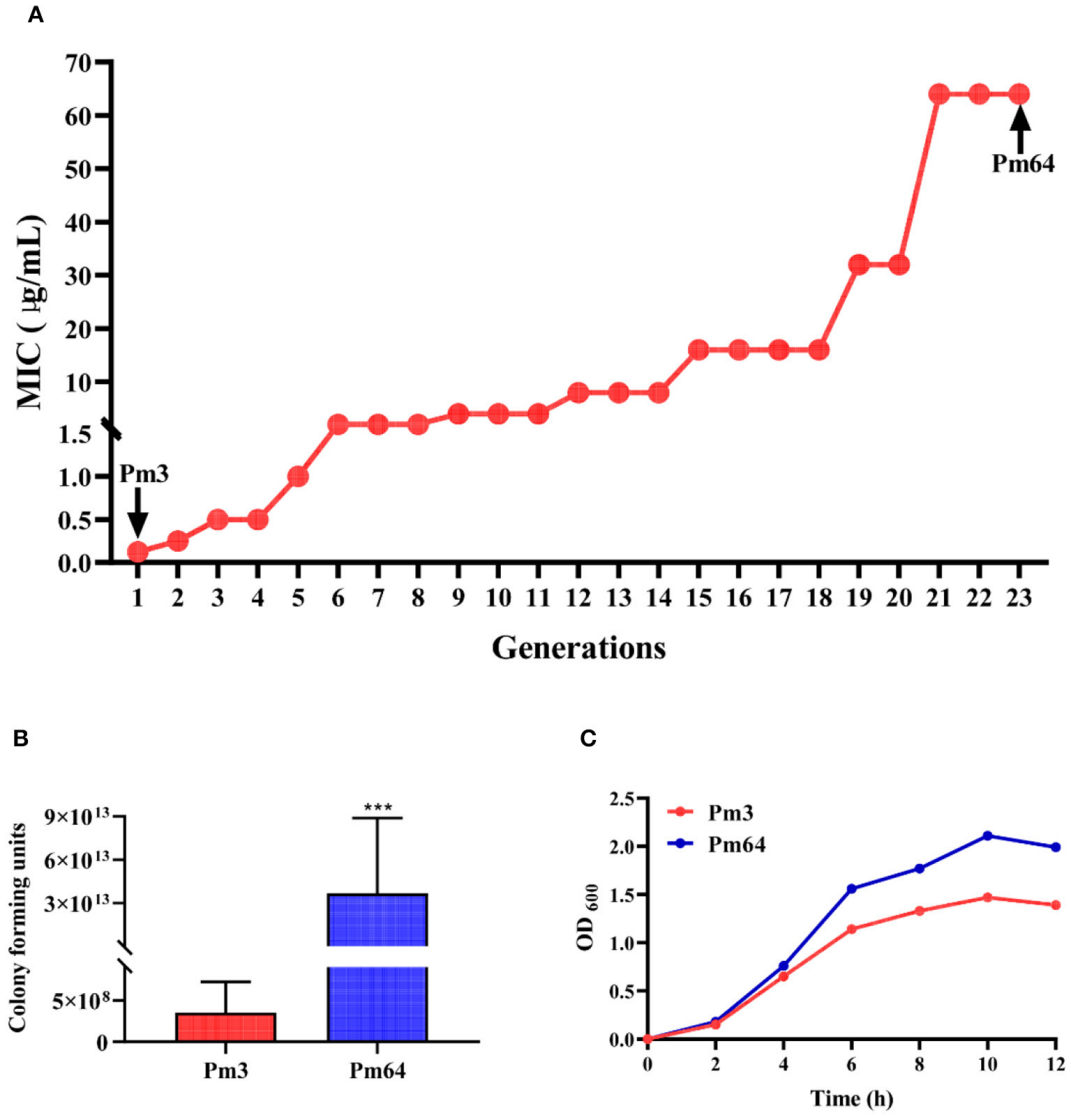
Biological characteristics of Pm3 and Pm64. **(A)** Induction process of Pm64 from Pm3 strain under continuous increasing subinhibitory concentration (1/2MIC) enrofloxacin culture condition. **(B)** LD_50_ of Pm3 and Pm64 to mice. **(C)** Growth curve of Pm3 and Pm64 (^***^p < 0.001).

Pm3 strain was isolated from the nasal cavity of cattle with BRD in the previous study (6). In this research, under the stimulation of a gradually increasing 1/2 MIC concentration of enrofloxacin culture environment, the MIC of enrofloxacin on Pm3 was increased from 0.05 to 64 μg/mL (continued culture for 21 generations *in vitro*). After more than two generations of continuous passage culture, Pm64 strain was obtained (Figure 1A).Then, the pathogenicity in mice (LD_50_) and culture characteristics *in vitro* of the two strains were examined (Figures 1B,C). Results showed Pm3 strain showed a strong virulence to mice (3.532 × 10^8^ CFU/mL per mice). Observing the pathological changes under a microscope, it can be seen that the alveolar wall capillaries are dilated; a large number of neutrophils infiltrate, which is consistent with the infection characteristics of P. multocida (Supplementary Figure 1). The LD50 for the Pm64 showed a significant reduction (3.682 ×1013 CFU/mL per mice) with an increased growth rate and plateau peak concentration on the growth curve. The virulence of the Pm64 strain showed significant attenuation during the formation of quinolone resistance.

### Whole-Genome Features of Pm3 and Pm64

Based on the significant differences in biological characteristics of *P. multocida* serogroup A isolates P3 after being induced by fluoroquinolones, Pm3 and Pm64 were selected for whole-genome sequencing to elucidate potential mechanisms of virulence attenuation. Circos circular representation of the Pm3 and Pm64 genome with annotated genes were constructed (Supplementary Figures 2A,B). Lengths of the obtained genomes were, respectively, 2,386,471 bp (Pm3) and 2,424,216 bp (Pm64) and contained 2,265 (Pm3) and 2,378 (Pm64) predicted coding genes. The GC content of the Pm3 and Pm64 genome was 40.28 and 40.3%, respectively, which showed a higher similarity (Supplementary Table 1). The complete nucleotide sequence of Pm3 and Pm64 sequenced in this study were submitted to GenBank with accession number CP081486 and CP081487.

### Gene Islands Comparison

**Figure 2:**
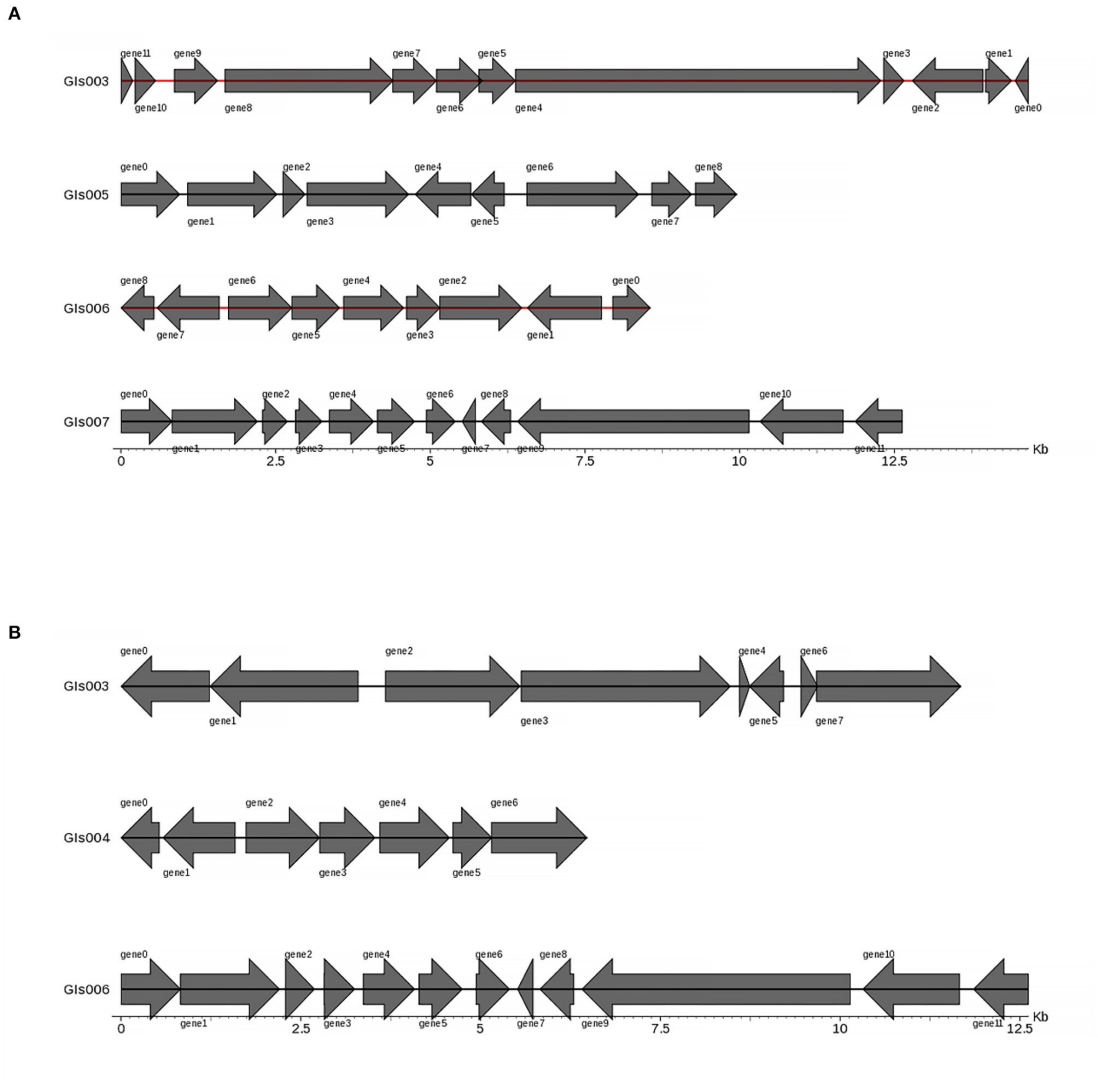
Statistical representation of gene distribution in GIs of Pm3 and Pm64. **(A)** Predicted GIs structure in Pm3 chromosome. **(B)** Predicted GIs structure in Pm64 chromosome. The horizontal coordinates are length scales and the lengh of gene islands shown in the figure are <15 kb.

**Figure 3:**
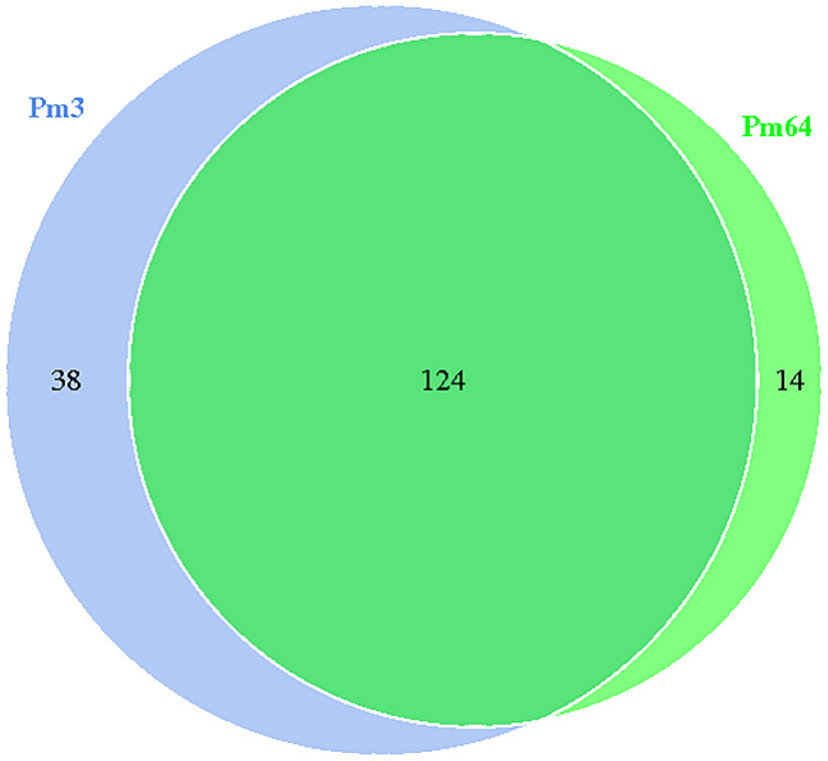
Venn diagram of core and specific gene cluster of Pm3 and Pm64 gene islands. Each ellipse represents a sample, the data on each region represents the groups number occur in and only in the samples. A group represents a set of genes with more than 50% similarity and sequence length difference < 0.7.

Gene islands (GIs) contribute to lateral gene transfer and bacterial evolution. Thus, the phylogenetically biased and mobility genes (such as transposases or integrases) of the Pm3 and Pm64 genome were detected to determine the GIs and the potential horizontal genes transfer. Eightand seven GIs were predicted in Pm3 and Pm64 genome, respectively (Table 1; Figure 2). Then population analysis was performed to analyze core and specific genes, and 38 and 14 genes were recognized as specific genes of Pm3 and Pm64 GIs (Figure 3); 24 and 8 genes of these could be realized with a potential function. We found some specific genes changed in the composition of GIs; those specific genes were involved in DNA binding, trehalose metabolism, material transportation, capsule synthesis, prophage, amino acid metabolism, and other functions (Supplementary Tables 3, 4).

**Table 1:** Gene islands (GIs) prediction results statistics.

**Sample**	**Pm3**	**Pm64**
GIs number	8	7
GIs total length (bp)	148,520	131,993
Average length (bp)	18,565	18,856
Predicted genes	162	138

### Genomic Annotation and Annotation Comparison

**Figure 4:**
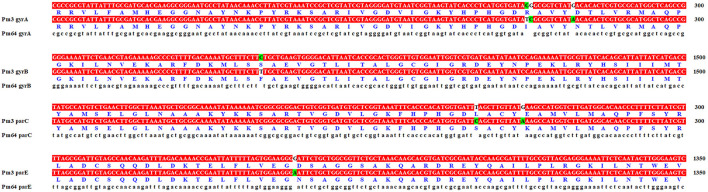
Quinolone resistance determining region (QRDR) comparison of Pm3 and Pm64. Only part of the base mutations sequence (labeled in green) was shown.

The genome sequencing results were compared and annotated with GO, KEGG, and COG databases. The annotation results between Pm3 and Pm64 strains were compared (Supplementary Figures 3–5). It was found that no new genes were obtained during the formation of enrofloxacin resistance in Pm64. Although there are numerical differences in the annotated results, most of them are the recurrence of some genes. Similarly, comparing annotations results from VFDB, ARDB, and CARD database, no new genes were obtained in Pm64. In order to explain the causes of the formation of fluoroquinolone resistance in Pm64, the quinolone resistance determining region (QRDR) genes *gyrA, gyrB, parC*, and *parE* were selected for sequence alignment (Figure 4). The results showed that all four genes had partial mutations of the QRDR sequence but only three gene mutations led to the replacement amino acid mutations (GyrA R88I, GyrB S467F, and ParC L84S).

### RNA-Seq Analysis and DEGs Identification of Pm3 and Pm64

**Figure 5:**
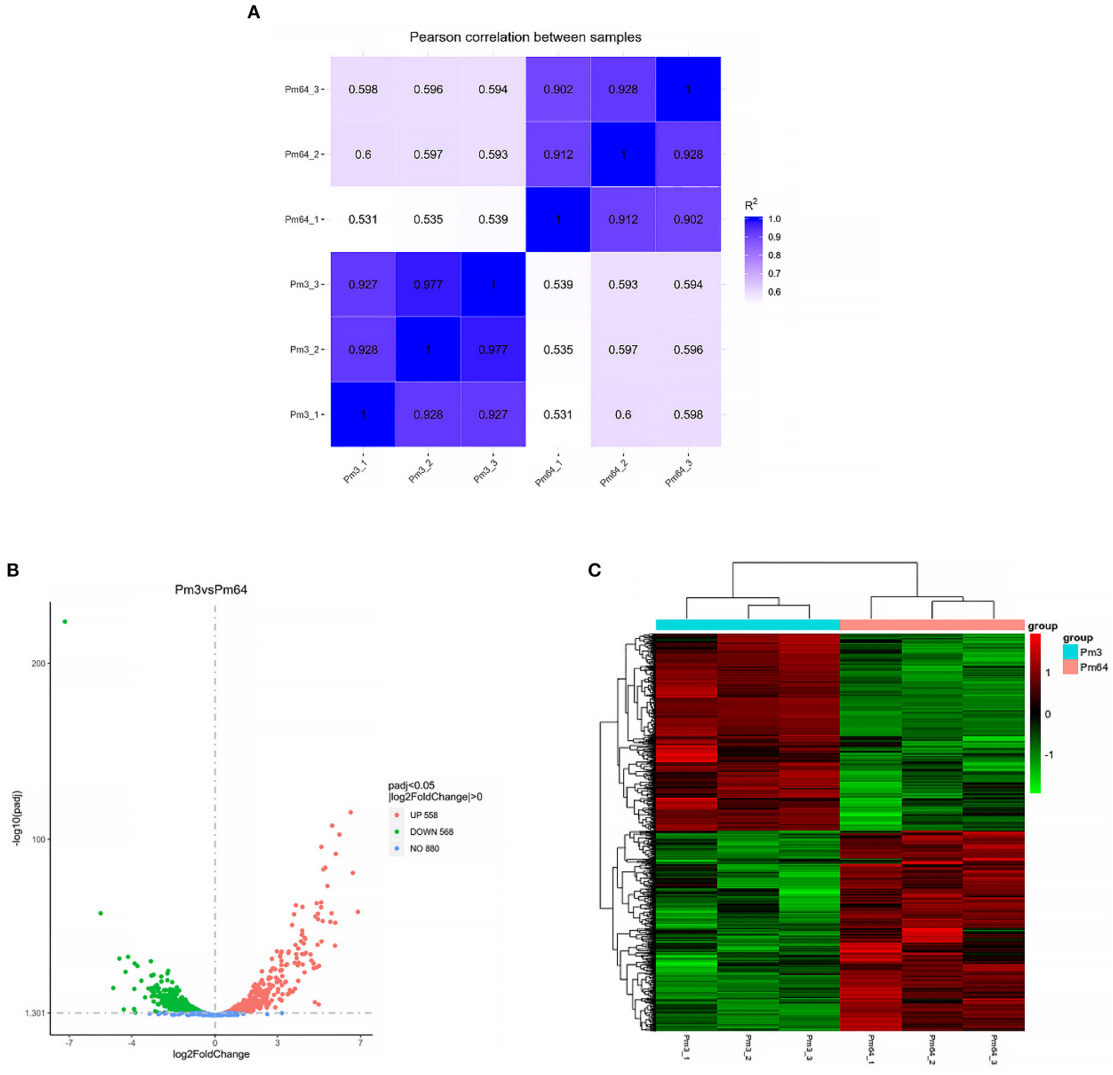
RNA-Seq analysis of Pm3 and Pm64. **(A)** Correlation map of gene expression. **(B)** Differential analysis volcano map. **(C)** The clustering heat map of DEGs.

**Figure 6:**
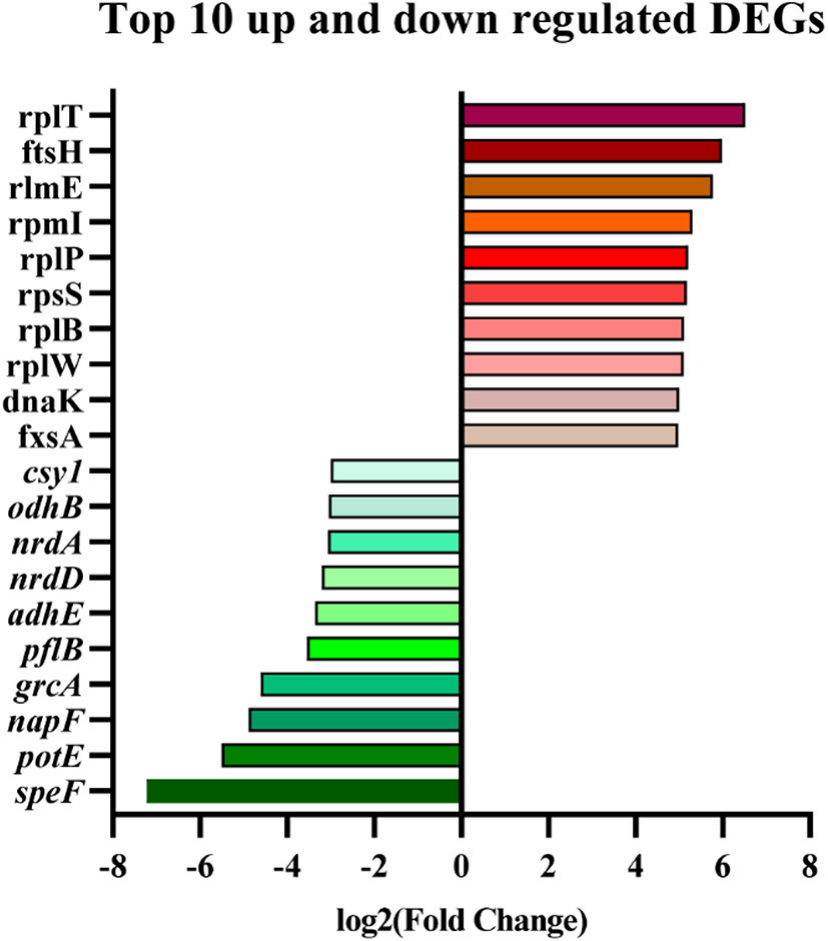
Top 10 up and down regulated DEGs screened from Pm3/Pm64.

All data on the quality of RNA-Seq are shown in Supplementary Table 5. The average percentages mapped to the reference genome were all above 98%. The correlation analysis of gene expression patterns (Figure 5A) showed significant differences among samples and good repeatability between groups (*R*^2^ > 0.9). The gene expressions' data were statistically analyzed to screen the genes with significant change in their expression in the samples of different states, and the significance of each gene in all comparison combinations was assessed by *P*-value test and FDR (False discovery rate) correction. Finally, a complete set of differential genes were identified. Based on the transcriptome analysis of Pm3 and Pm64 [log2(FoldChange)| > 0 & Padj < 0.05], a total of 1,126 DEGs were observed, including 558 up-regulated and 568 down-regulated genes (Figures 5B,C). The top 10 up and down regulated DEGs were selected and shown in Figure 6.

### Virulence Related DEGs of Pm3 and Pm64

**Figure 7:**
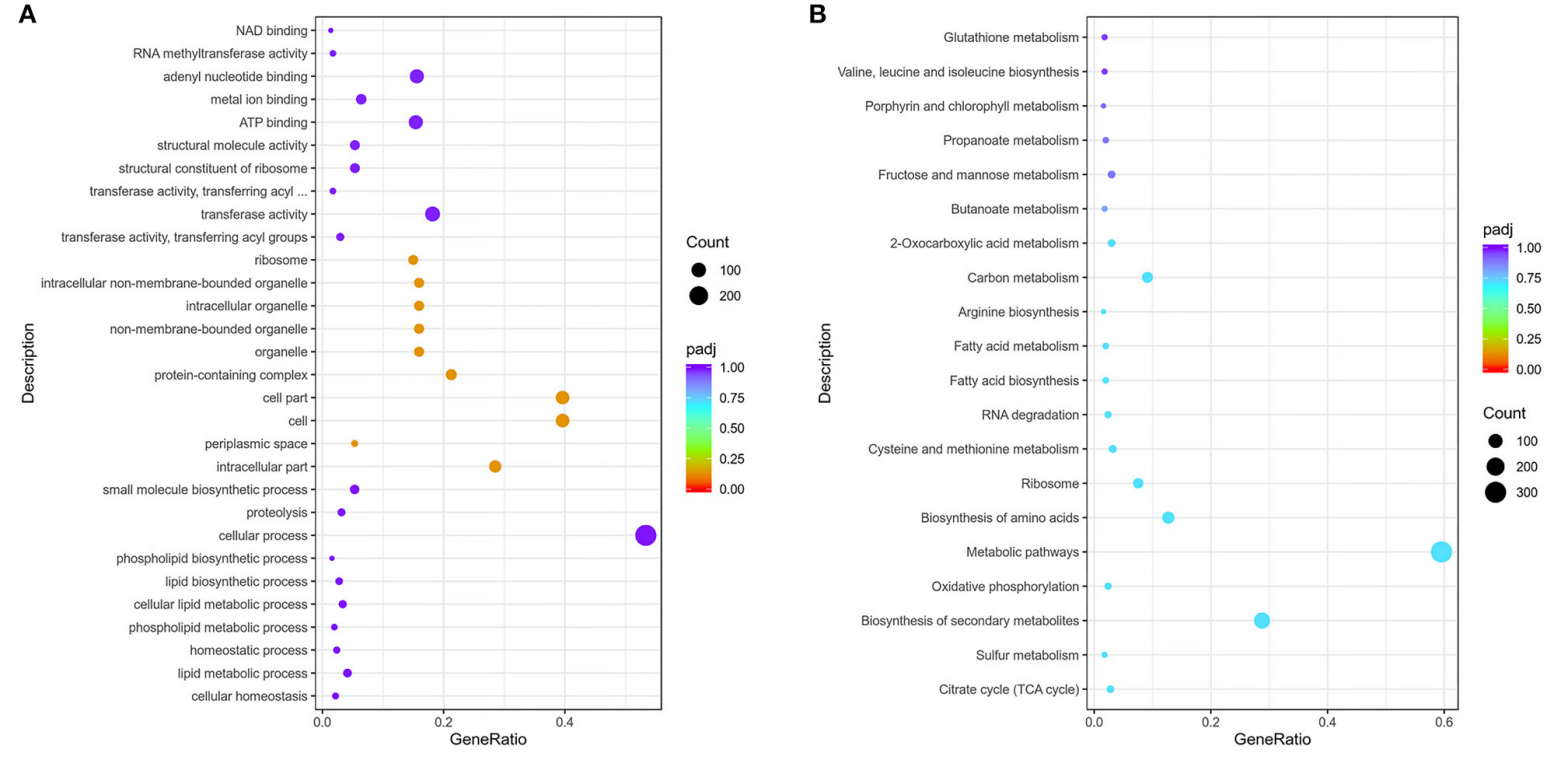
Scatter plot of GO and KEGG enrichmen. **(A)** Top 30 enriched GO function and DEGs numbers enriched in each function of Pm3/Pm64. **(B)** Top 20 enriched KEGG pathways and the number of DEGs in of Pm3/Pm64. The abscissa represents the ratio of DEGs annotated to the total differential genes; the ordinate represents the GO Term and KEGG pathway, respectively; the size of the point represents the number of annotated genes; the color from red to purple represents the decreasing significance of enrichment.

Functional enrichment of filtered DEGs of the virulent Pm3 strain and virulence attenuation Pm64 strain were carried out based on GO and KEGG database. By GO enrichment analysis, 508 DEGs were matched to three parts of the gene function: BP (Biological Process), CC (Cellular Component), and MF (Molecular Function). The top 30 enriched GO functions was shown in Figure 7 and Supplementary Figure 6. In the overall DEGs function enrichment, CC is the most enriched, mainly including associated functions of cell, intracellular, organelle, periplasmic space, and ribosome. However, the up-regulated DEGs were more concentrated in BP, mainly nitrogen, sulfur, phosphorus metabolism, and phospholipid biosynthesis. At the same time, the down-regulated genes were concentrated in MF, which was mainly reflected primarily in the weakened activities of some transferases, oxidoreductases, kinases, and some molecules' binding ability. By KEGG enrichment analysis, a total of 504 DEGs were identified. The top 20 significantly enriched KEGG pathways were shown in Figure 7B, including Citrate cycle (TCA cycle), Sulfur metabolism, Biosynthesis of secondary metabolites, Oxidative phosphorylation, Metabolic pathways, Amino acids biosynthesis and metabolism, Ribosome, RNA degradation, Fatty acid biosynthesis and metabolism, Carbon metabolism, 2–Oxocarboxylic acid metabolism, Butanoate metabolism, Fructose and mannose metabolism, Propanoate metabolism, Porphyrin and chlorophyll metabolism, and Glutathione metabolism. Moreover, 20 virulence factors annotated in VFDB and related to five lipopolysaccharide and lipooligosaccharide (LPS/LOS), four pilis (including Flp pili and Type IV pili), five iron utilization, and six others were significantly changed in the formation of virulence attenuated Pm64 strain (Supplementary Table 6).

**Figure 8:**
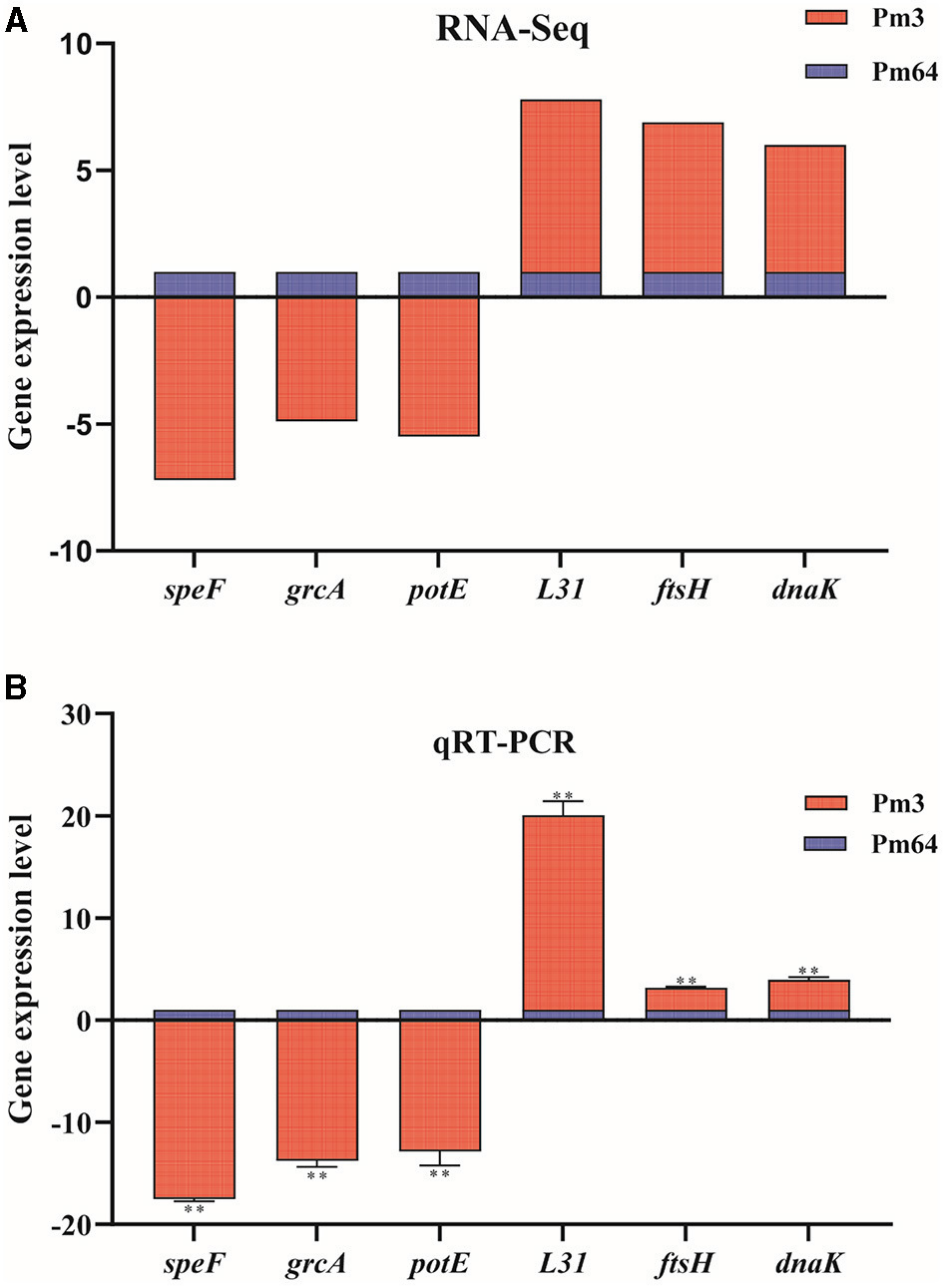
Expression level validation of part of virulence factor-related DEGs (*n* = 3). **(A)** The mRNA expression (RNA-Seq) of virulence factor-related DEGs in Pm3/Pm64. **(B)** The mRNA expression (RNA-Seq) of virulence factor-related DEGs in Pm3/Pm64. Results were representative of two independent experiments, with three replicates per group, analyzed by multiple comparative analysis and expressed as means ± SD (^*^p < 0.05, ^**^p < 0.01).

To validate the transcriptomic analysis results, three significantly down-regulated genes (*speF, grcA, potE*) and three significantly up-regulated genes (*L31, ftsH, dnaK*) were selected for qRT-PCR validation. The relative expression levels(2^−Δ*ΔCt*^) of these genes with the transcriptome quantification results (log2FoldChange) were shown together in Figure 8, which shows a consistent trend from qRT-PCR results to RNA-seq results.

## Discussion

Based on previous studies, *P. multocida* serogroup A is considered the major pathogen of BRD (2, 19, 20). The persistent respiratory tract infection caused by *P. multocida* serogroup A has always been a huge issue for the cattle industry. The prevention of and treatment measures for *P. multocida* are still based on broad-spectrum antibiotics, but the increasing use of antibiotics (such as fluoroquinolones and macrolides) has led to the increase of resistance and resistance spectrum of *P. multocida* (6). In view of this situation, while standardizing antibiotics, new strategies should be sought to slow down the emergence of *P. multocida* resistance and effectively suppress the harm of *P. multocida* to the host. While much progress has been made in understanding bacterial resistance and virulence development, major gaps remain in our knowledge of the potential adaptive evolution of virulence and resistance mechanisms. We used global transcriptome and genome analyses to address theses gaps to identify the underlying regulatory and genetic adaptations in acquired fluoroquinolone-resistant *P. multocida* serogroup A. The results of our study revealed the multiple pathways in DNA replication recombination and repair, amino acid transport and metabolism, inorganic ion transport, and cell wall and membrane biogenesis that is associated with the attenuation of virulence following acquired resistance of *P. multocida* serogroup A.

The original intention of the study was to simulate the formation of fluoroquinolone resistance of *P. multocida* serogroup A *in vitro* to analyze the genetic changes in the development of resistance phenotypes in *P. multocida*. Then the study aimed to find the target of antibiotic resistant inhibitors that can be used in the follow-up screening of synergistic drug use with antibiotics.

Resistance mutations often map to genes that encode antibiotics targeted proteins; these proteins are mostly involved in some essential functions such as DNA replication, transcription and translation, or cell wall biosynthesis (21). The mutations that endowed resistance to antibiotics often provide a selective advantage in the presence of a hostile environment, but these mutations tend to alter the biochemistry of the target protein, which will adversely affect the biological function of mutants, sometimes resulting in reduced fitness of the microorganism (also known as fitness costs) (22, 23). However, in some cases, compensatory mutations ameliorate fitness costs of resistance mutations from secondary mutation, gene duplication, ectopic dominance, and metabolic compensation (12, 24–27). It was suggested that fitness costs and compensatory evolution occurred in fluoroquinolone resistance acquisition in Pm64, which allowed us to move beyond the target mutation from quinolone resistance to the mechanisms of virulence attenuation due to acquired resistance. Thus, the whole genome sequences and transcriptome expression profiles related to highly virulent quinolone sensitive strain Pm3 and virulent attenuated quinolone-resistant strain Pm64 are explored in this study.

Resistance development against fluoroquinolones can occur via various mechanisms ranging from chromosomal gene mutations to the specific transferable genes acquisition (28). The mutations in the chromosomal elements encoding the target enzyme (quinolone resistance determining region, QRDR) DNA gyrase (*gyrA, gyrB*) and topoisomerase IV *(parC, parE*) can considerably alter the susceptibility of the isolates, while the acquisition specific genes are always involved in overexpression of efflux pumps, alteration of the membrane permeability, and the expression of inactivation enzymes (28–30). Since foreign plasmids were not acquired in the genome, the acquired horizontal transfer of quinolone resistant genes was excluded. Only chromosome-mediated spontaneous mutations of QRDR genes were detected in this study. The complete ORF genes (*gyrA, gyrB, parC*, and *parE*) of Pm3 and Pm64 were compared by sequence alignment. It was shown from comparison that the QRDR genes had incurred varying degrees of base mutation in Pm64 induced from Pm3, which leads to the amino acid substation GyrA R88I, GyrB S467F, and ParC L84S (Figure 5). It suggests that these site mutations may be the vital cause of quinolone resistance of *P. multocida* serogroup A, which requires further in-depth verification.

Genomic islands (GIs) of prokaryotes are discrete inserted DNA segments obtained by horizontal transfer which carries genes that could affect pathogenicity, antibiotic resistance, metabolism, and adaptability (31–33). Compared with Pm3, the GIs of Pm64 had lost some genes involved in trehalose metabolism (*treB, treC* and *treR*). They added several capsular polysaccharide syntheses and transport-related genes (*lipA, lipB*) and a gene *folD* that mediates tetrahydrofolate metabolism. Trehalose is a sugar widely distributed in bacteria, fungi, and other higher organisms, playing different biological functions. In some plant bacterial pathogens, it plays a important role in enhancing colonization and enhancing virulence (34, 35).The trehalose-monomycolate is a precursor for the synthesis of mycolic acid essential for the synthesis of the bacterial cell wall and is pathogenic (36). Capsule plays the most critical role of *P. multocida* serotype A, which is a critical structural component and a virulence factor (37). Hyaluronic acid (HA) is a component of some types of capsules of *P. multocida serotype A*, which endows the strain with anti-phagocytosis ability and the bactericidal action defense from antimicrobial agents (38, 39). FolD occupies a central position in the folate-dependent C1 metabolism, and the folate-dependent C1 metabolism provides the key building blocks for growth, most importantly nucleic acids, amino acids, provitamines, and formylated methionine tRNA for translation initiation (40, 41). Therefore, we speculated that these genetic changes in GIs were of great significance for virulence attenuation, fluoroquinolone resistance, and growth performance enhancement of *P. multocida* serotype A.

In microbial infections, the virulence genes are the major determinants of disease severity (33). In explaining the mechanism of Pm64 virulence attenuation, more attention should be attached to the level of gene expression in addition to the presence or absence of virulence genes. Therefore, the differences in the expression levels of virulence factor related genes were analyzed in transcriptome sequencing results.

Fimbrial low-molecular-weight protein (Flp) pili are assembled and secreted by a complex of proteins encoded by the tad operon,which plays an important role in the establishment and resistance of *Vibrio vulnificus* biofilms to mechanical clearance (42). The Type IV pili, meanwhile, contributes to biofilm formation and auto agglutination (43). It was reported that the amount of capsular polysaccharide (CPS) produced by *P. multocida* is inversely proportional to the amount of biofilm formed. Through mutation or *in vitro* passage, CPS can be lost or reduced. Finally the deficient biofilm-forming strains can be transformed into robust biofilm-forming strains (32). Four pili-related genes (*tadD, tadF, comE*, and *flp1*) were up-regulated in Pm3/Pm64, which increased in biofilm formation and may lead to the decrease of CPS; in turn this leads to the attenuation of virulence to a certain extent.

Lipid A-anchored lipopolysaccharide (LPS) or lipooligosaccharide (LOS) in the outer leaflet of the outer membrane are also major virulence factors of *Gram-negative bacteria*, which are essential for bacterial viability and fitness in the host (44). And the inflammatory response to the endotoxic lipid A component is a significant cause of infection pathogenesis (45). There are two up-regulated (lpxB and lpxC) and three down-regulated (galE, rfaC, and msbA) LPS related genes in RNA Sequencing. In Gram-negative bacteria, LpxB and LpxC are essential for the lipid A biosynthesis, so maintaining the structure of the bacterial cell envelope and its growth (44, 46, 47). GalE was associated with exopolysaccharides (EPS) and LOS. The disruption of *galE* gene in *Glaesserella parasuis* could produce more biofilms and increase the sensitivity to porcine serum (48). RfaC is necessary for oligosaccharide synthesis, which is a core block of LPS formation. It was involved in flagella assembly, T3SS secretion mechanism, and protein secretion (49). Some studies have reported that it is related to the fitness cost of *E. coli* (50, 51). LPS ABC transporter MsbA is associated with the transbilayer movement of lipid A-core molecules from the cytoplasmic to the periplasmic face of the inner membrane,which is also a putative determinant of tetracycline resistance (52, 53). The changes in expression levels of LPS and LOS related gene in Pm64 not only affect the host viability and antibiotics adaptation mediated by LPS and LOS, but also greatly affect the biofilm and potentially attenuate CPS production.

Iron assimilation and its utilization are crucial for the cells' biological functions, which are not only involved in pathogenesis but also the resistance of strains (54). For pathogenic bacteria, heme is the major source of nutritional iron. Heme biosynthesis is an important cofactor of peroxidases, catalases, sensor molecules, and cytochromes, indirectly coupled to respiration (55). For the DEGs associated with heme biosynthesis, *hemG* (protoporphyrinogen oxidase), *hemH* (ferrochelatase), and *hemE* (uroporphyrinogen decarboxylase) were down-regulated and two heme transportation related genes (*ccmB* and *ccmE*) were up-regulated in Pm3/Pm64. It suggests that iron assimilation and its utilization may be the key mechanisms for the formation of quinolone resistance and the resulting attenuation of virulence.

The major outer membrane protein DnaK plays a key role in native protein folding, which was also recognized as a potential therapeutic targets in *Mycobacterium smegmatis* and *Helicobacter pylori* (23, 56). Other studies have shown that *dnaK* enhances the virulence of *Listeria monocytogenes* (57). Compared with Pm64, the *dnaK* gene in Pm3 was the most significantly up-regulated among all the screened virulence factors' DEGs. This indicates that *dnaK* may play an essential role in stress survival and virulence attenuation in *P. multocida* serogroup A.

In addition, we found some special genes that might influence the virulence but are not annotated toVFDB. These include *speF* (ornithine decarboxylase), *potE* (putrescine-ornithine antiporter), and *ftsH* (Atp-dependent zinc metalloproteinase). The polyamines have been described as key signals of virulence in pathogenic bacteria. Putrescine is the most important polyamine in bacterial cells, which could be synthesized by ornithine decarboxylation of redundant enzymes encoded by the *speC* and *speF* genes (58). The *potE* gene encodes a membrane protein that was associated with exchange reactions of putrescine and ornithine (59). The *speF* and *potE* are the two most obvious down-regulated genes in Pm3/Pm64. It is speculated that they may have a negative regulatory effect on pathogenicity. FtsH is a membrane protease that is critical for degrading membrane proteins. The decrease of proteolytic activity of FtsH protease could promote the pathogenicity of *Salmonella* in phagocytic cells, as well as the negative effects on growth, stress tolerance, and biofilm formation on *Lactobacillus plantarum* (60, 61). Moreover, the overexpression of *ftsH* in *Mycobacterium tuberculosis* causes growth retardation (62). These phenotypes were consistent with what we observed in Pm3/Pm64, suggesting an important role of these genes in the virulence attenuation mechanism of *P. multocida* serogroup A. Furthermore, some 30/50s ribosome proteins were down-regulated in Pm64 strain, suggesting more translational modification changes in the mechanism of *P. multocida* virulence attenuation, which needs more in-depth studies in the future.

While deeply exploring the mechanism of *P. multocida* virulence attenuation, an important question that needs to be discussed is whether virulence-virulence reduced strains can revert to a highly virulent phenotype if they infect animals and lose the inhibition of drugs for a long time. Similar results were not found in our study, and may require more *in vivo* data.

In conclusion, the study showed that, after being induced by enrofloxacin at the subinhibitory concentration, the virulence of the *P. multocida* serogroup A resistant strain decreased significantly. The potential mechanisms may be related to the loss genes of GIs at the genomic level and the expression changes of some virulence and drug-resistance related genes at the transcriptional level. Several candidate genes that may be highly important for the virulence attenuation of *P. multocida* serogroup A strains were found in this study, which may facilitate the design of new and improved vaccines. The data presented here provide fundamental background knowledge that would help follow-up research on pathogenesis and antimicrobial drugs development.

## Data Availability Statement

The datasets presented in this study can be found in online repositories. The names of the repository/repositories and accession number(s) can be found below: https://www.ncbi.nlm.nih.gov/genbank/, CP081486; https://www.ncbi.nlm.nih.gov/genbank/, CP081487; https://www.ncbi.nlm.nih.gov/, GSE182406.

## Author Contributions

LZ and JZ: collected the samples. LZ, JZ, BZ, and XL: performed the experiments. RH, LK, XZ, LZ, and JZ: wrote the manuscript. All authors read and approved the final manuscript.

## Funding

This research was supported by the National Natural Science Foundation of China Youth Fund (31702293) and the China Agriculture Research System of MOF and MARA (CARS-37).

## Conflict of Interest

The authors declare that the research was conducted in the absence of any commercial or financial relationships that could be construed as a potential conflict of interest.

## Publisher's Note

All claims expressed in this article are solely those of the authors and do not necessarily represent those of their affiliated organizations, or those of the publisher, the editors and the reviewers. Any product that may be evaluated in this article, or claim that may be made by its manufacturer, is not guaranteed or endorsed by the publisher.
