# Supplementary material for: Genomic and Transcriptomic Analysis of Bovine Pasteurella multocida Serogroup A Strain Reveals Insights Into Virulence Attenuation
Source: Front Vet Sci. 2021 Nov 10;8:765495. doi: 10.3389/fvets.2021.765495 (PMC8631534; doi:10.3389/fvets.2021.765495)
Supplement: Supplementary file 1 [file Data_Sheet_1.pdf]

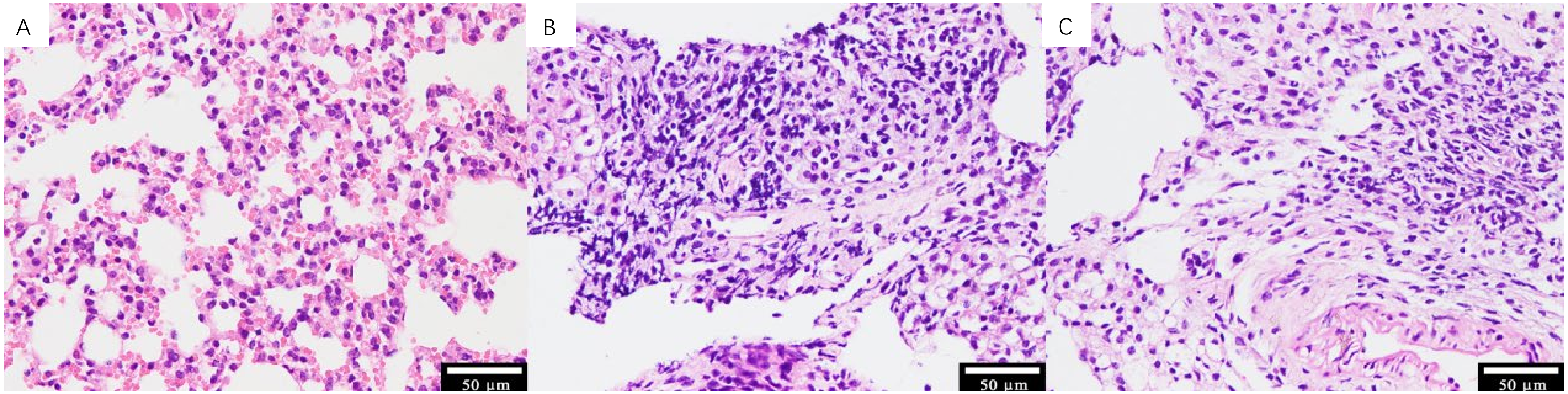

Supplement Figure S1. Observation of Lung Pathological Section. A: PBS control group; B: Pm3 challenge group. All images scale bars are 50 μm.

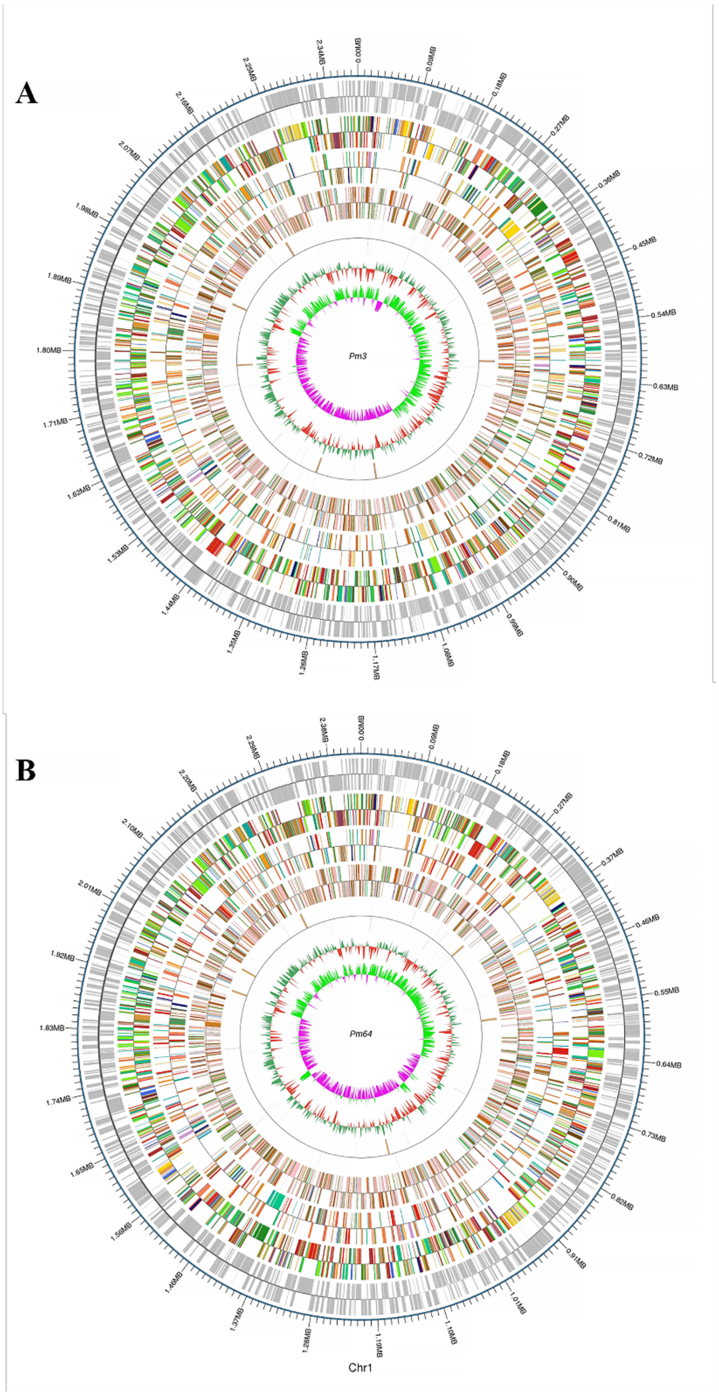

Supplement Figure S2. Relevant genomic features of Pm3 and Pm64 chromosome. (A) Relevant genomic features of Pm3 chromosome. (B) Relevant genomic features of Pm64 chromosome. Moving inward from the outermost circle, each ring of the circle contains information of a genome: coding genes, gene function annotation results (include the annotation results information of COG, KEGG, GO databases), ncRNA, genome GC content and Genomic GC. The inward red part of genome GC content indicated the lower GC content of the whole genome in this region, while the outward green part was contrary. The higher the peak value shows the greater difference between the GC content and the average GC content. The pink part inward of the Genomic GC skew value indicates that the content of G in this region is lower than C, and the light green part outward is contra.

Supplement Table S1. qRT -PCR primers used in this work

Genes	Primers
ftsH	F：TGTTCCGTTTCTACCGCACCTTTC<br>R：CGTCAACCTGTTACACCGCCATC
dnaK	F：GCGAAAGGTGAAGATAAAGCGGAAATC<br>R：GCATCCACCACATCATCATTTGACTTAC
L31	F：CCAGAACCACAACGACCATGAAATG<br>R：TAGTACGGGTGTGATGCTGAGGAG
speF	F：GCTCGGCACTTATGACGGAACC<br>R：ACCTACCCACGCAGAATCAAACAAG
potE	F：GGGATAACGTTCGTGACGACGG<br>R：GTAAGCCTCTCCCCTGTTGTGATC
grcA	F：CGTACAGCGTAACCTGAAACACGG<br>R：CGCGAAACCTTAGAAGATGCGGTC
16S rRNA	F: GCCACGTCTCAAGGACACAG<br>R: TGGGGAGCAAACAGGATTAGA

Supplement Table S2. Genomic characteristics of Pm3 and Pm64

Sample	Pm3	Pm64
Type	Chromosome	Chromosome
GC%	40.28	40.3
Genome size(bp)	2,386,471	2,424,216
Gene number	2,265	2,328
Gene length/Genome(%)	89.01	89.07
Gene total length(bp)	2,124,156	2,159,325
Gene average length(bp)	938	928

Supplement Table S3. Specific gene of predicted gene islands in Pm3

No	Gene annotations	Potential function
1	GO:0006352	DNA-dependent transcription initiation
2	GO:0016887	ATPase activity
3	*RecT*	DNA binding, DNA metabolic process
4	COG3617	Prophage anti repressor X Mobilome
5	*MinE*	Septum formation topological specificity factor
6	COG3657	Putative component of the toxin-antitoxin plasmid stabilization module
7	COG3636	DNA-binding prophage protein X Mobilome
8	GO:0043565	Sequence-specific DNA binding
9	*rpiR*	sequence-specific DNA binding transcription factor
10	*YozV*	Uncharacterized membrane protein
11	GO:0003899	DNA-directed RNA polymerase activity
12	COG3617	Prophage anti repressor X Mobilome
13	COG4723	Phage-related protein, tail component X Mobilome
14	*treR*	LacI family transcriptional regulator, trehalose operon repressor
15	*treB*	Phosphotransferase system (PTS), trehalose specific IIB component
16	COG2315	Predicted DNA-binding protein
17	*treC*	Trehalose-6-phosphate hydrolase
18	*YhaK*	Redox-sensitive bicupin
19	*chrR*	NQR chromate reductase, NAD(P)H dehydrogenase (quinone)
20	*kefB*	Glutathione-regulated potassium-efflux system protein
21	*ccmA*	Heme exporter protein A; Membrane transport
22	*ccmB*	Heme exporter protein B; Membrane transport
23	*ahpC*	Peroxiredoxin (alkyl hydroperoxide reductase subunit C)
24	*metC*	Cysteine-S-conjugate beta-lyase

Supplement Table S4. Specific gene of predicted gene islands in Pm64

No	Gene annotations	Potential function
1	*KpsS; lipB*	Capsule polysaccharide modification protein
2	*kpsC, lipA*	Capsule polysaccharide modification protein
3	COG1196	Chromosome segregation ATPase
4	*FcbD*	Chondroitin synthase; Glycan biosynthesis and metabolism
5	GO:0003677	DNA binding
6	*sfsB*	Ner family transcriptional regulator
7	GO:0006313; GO:0015074	DNA-mediated transposition; Bacteriophage Mu transposase
8	*folD*	methylenetetrahydrofolate dehydrogenase (NADP+)/methyltetrahydrofolate cyclohydrolase

A

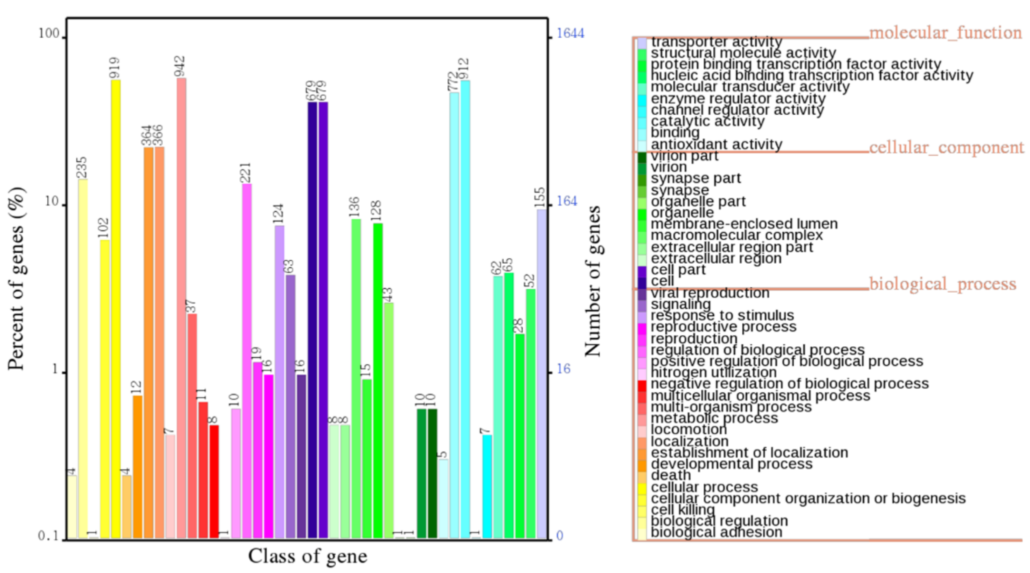

B

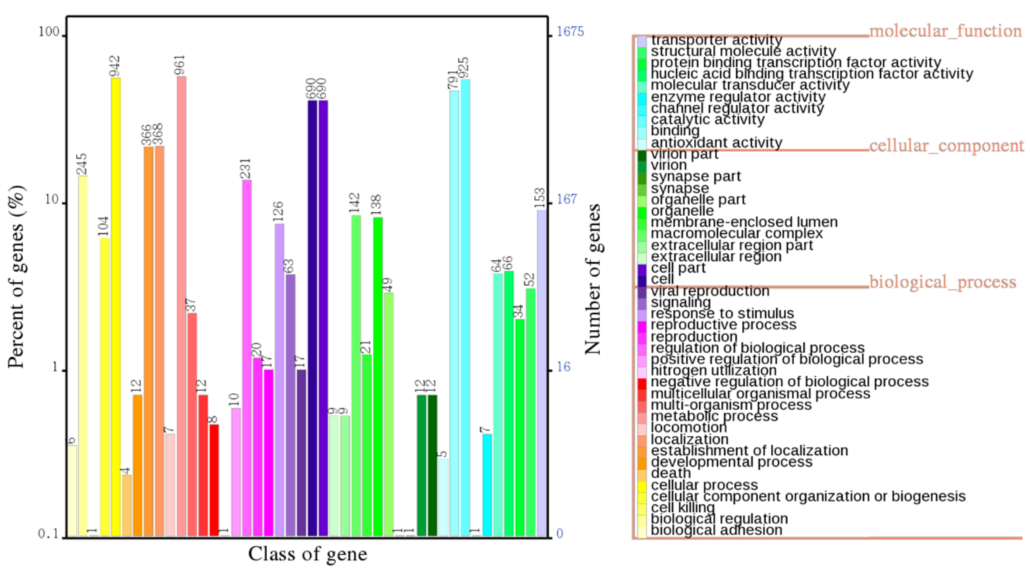

Supplement Figure S3. GO functional classification of Pm3(A) and Pm64(B) genome. The horizontal coordinate represents the GO functional classification on the sample annotation, the vertical coordinate on the right represents the genes number on the annotation, and the vertical coordinate on the left represents the percentage of the number of genes on the annotation in all coding genes.

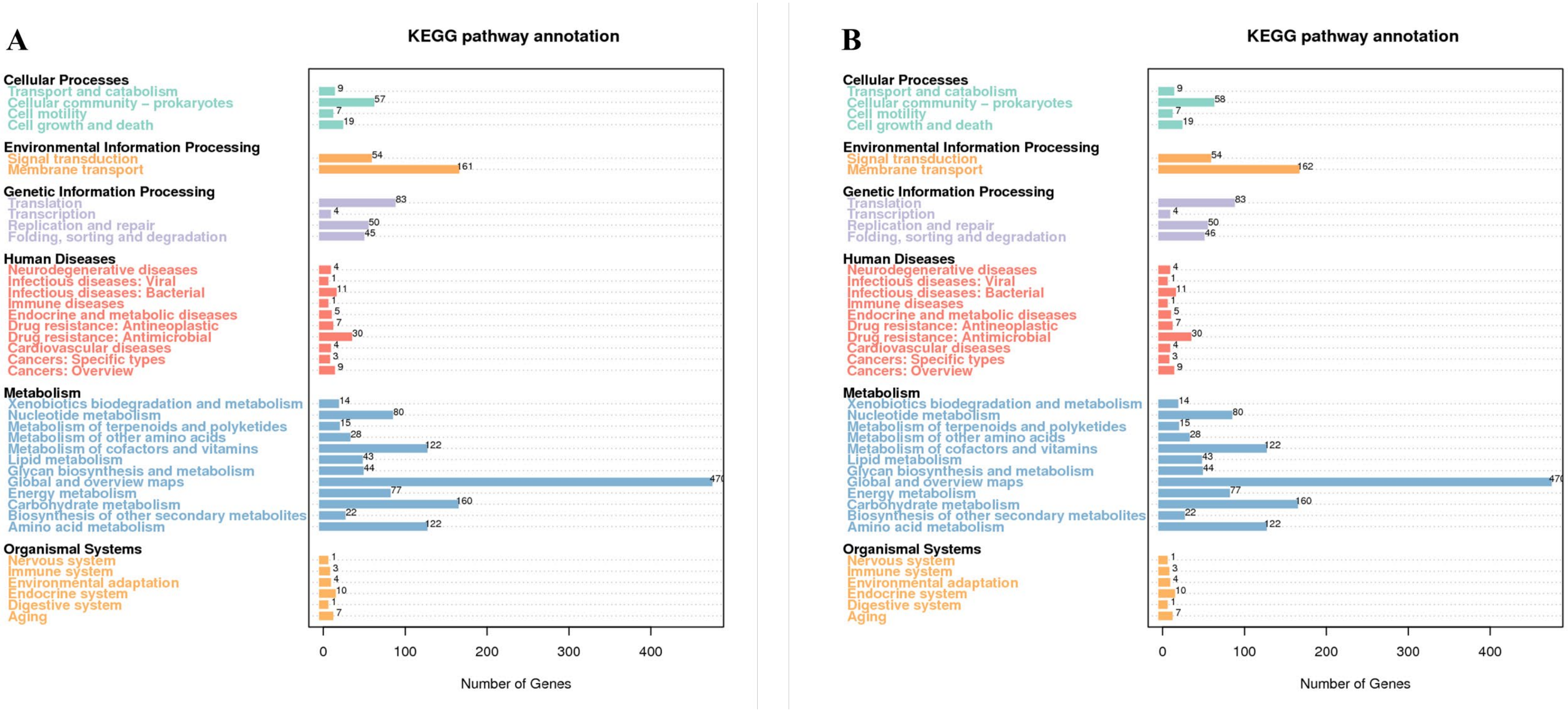

Supplement Figure S4. Classification of KEGG metabolic pathways of Pm3(A) and Pm64(B) genome. The numbers on the bar chart represent the annotated genes number. The remaining axis is the code for each level1 function class in the database.

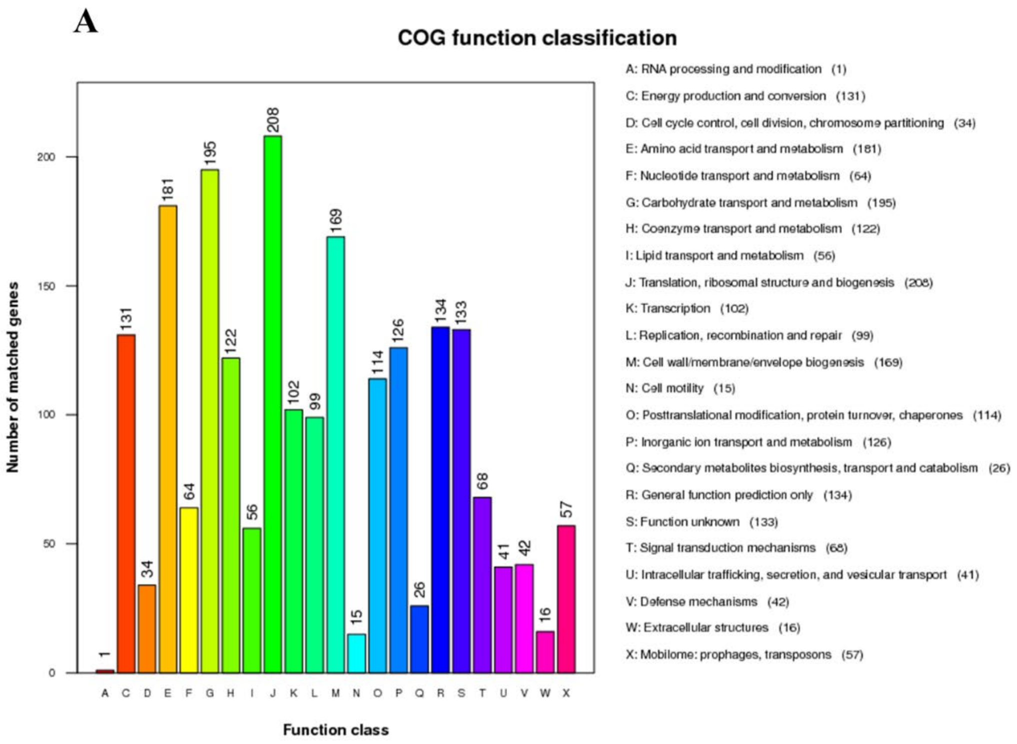

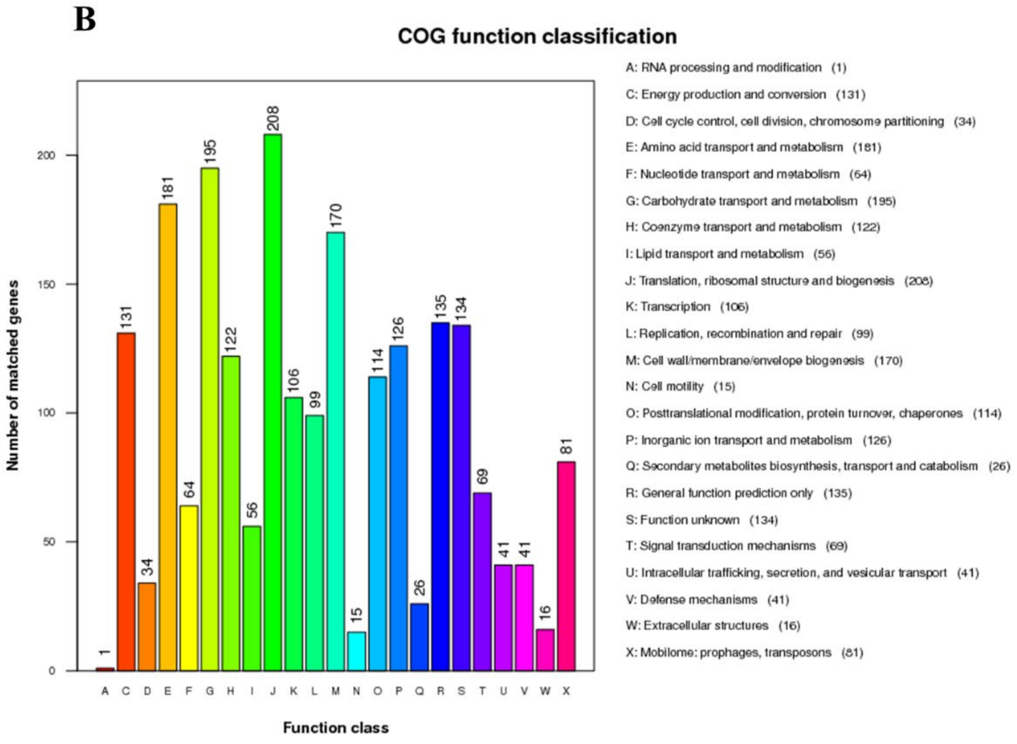

Supplement Figure S5. COG functional classification of Pm3(A) and Pm64(B) genome. The horizontal coordinate represents the functional type of COG, and the vertical coordinate represents the number of genes annotated.

Supplement Table S5. Transcriptome sequencing results statistics and quality assessment.

Sample	Pm3_1	Pm3_2	Pm3_3	Pm64_1	Pm64_2	Pm64_3
Total reads	13656988	13618462	15769964	15027712	14135486	16737618
Total mapped	13502751 (98.87%)	13446591 (98.74%)	15567568 (98.72%)	14782357 (98.37%)	13931429 (98.56%)	16424362 (98.13%)
Multiple mapped	125013 (0.92%)	128446 (0.94%)	176262 (1.12%)	193184 (1.29%)	158587 (1.12%)	216796 (1.3%)
Uniquely mapped	13377738 (97.96%)	13318145 (97.79%)	15391306 (97.6%)	14589173 (97.08%)	13772842 (97.43%)	16207566 (96.83%)
Positive map	6690632 (48.99%)	6660320 (48.91%)	7698061 (48.81%)	7297225 (48.56%)	6889401 (48.74%)	8108472 (48.44%)
Negative map	6687106 (48.96%)	6657825 (48.89%)	7693245 (48.78%)	7291948 (48.52%)	6883441 (48.7%)	8099094 (48.39%)
Reads mapped in proper pairs	13281818 (97.25%)	13220458 (97.08%)	15281178 (96.9%)	14494914 (96.45%)	13699712 (96.92%)	16115110 (96.28%)
Proper-paired reads map to different chrom	0 (0%)	0 (0%)	0 (0%)	0 (0%)	0 (0%)	0 (0%)

Total reads: clean data of sequencing filtered by sequencing data; Total Mapped: sequences could be mapped to the genome; Multiple Mapped: sequences with multiple alignments on the reference sequence; Redundant mapped: sequences with a unique alignment position on the reference sequence; Positive map, negative map: number of positive and negative sequences that have been aligned to the genome; Reads mapped in proper pairs, proper-paired reads map to different chrom: Paired sequence mapped to the reference genome and different chromosomes.

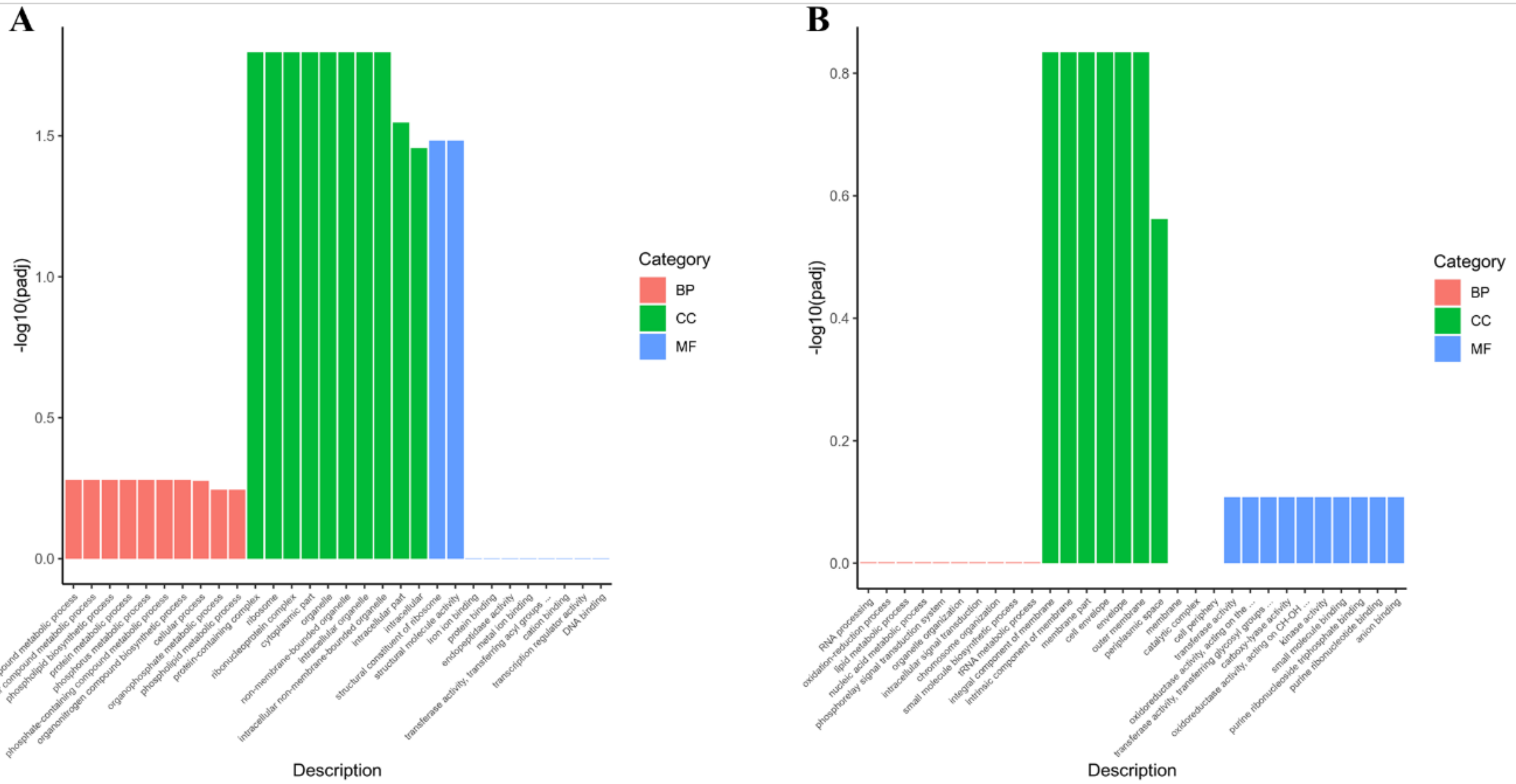

Supplement Figure S6. GO enrichment analysis of DEGs (Pm3 vs Pm64). (A). Functional enrichment of up-regulated genes. (B). Functional enrichment of down-regulated genes.

Supplement Table S6. Virulence factor related DEGs annotated in VFDB from Pm3/Pm64

No	VF_id	VF_name	Related_genes	Log2FoldChange
**up-regulated**				
1	AI392	MOMP	*dnaK*	5.00
2	AI104	fimbrial low-molecular-weight protein (Flp) pili	*tadD*	3.20
3	AI104	fimbrial low-molecular-weight protein (Flp) pili	*tadF*	3.14
4	VF0260	RelA	*relA*	2.27
5	CVF358	Cytochrome c muturation (ccm) locus	*ccmB*	1.86
6	CVF494	LPS	*lpxC*	1.69
7	CVF486	Type IV pili	*comE*	1.64
8	CVF358	Cytochrome c muturation (ccm) locus	*ccmE*	1.40
9	CVF494	LPS	*lpxB*	1.29
10	AI104	Flp Type IV pili	*flp1*	1.25
11	CVF309	Leucine synthesis	*leuD*	1.00
**down-regulated**				
12	CVF494	LOS	*galE*	-2.96
13	CVF494	LPS	*rfaC*	-2.83
14	CVF845	Pyrimidine biosynthesis	*carA*	-2.19
15	CVF506	Heme biosynthesis	*hemH*	-2.17
16	CVF494	LOS	*msbA*	-1.44
17	CVF491	OapA	*oapA*	-1.13
18	CVF506	Heme biosynthesis	*hemE*	-1.07
19	CVF506	Heme biosynthesis	*hemG*	-1.01
20	CVF308	Tryptophan synthesis	*trpD*	-1.00
